# Rational coordination regulation in carbon-based single-metal-atom catalysts for electrocatalytic oxygen reduction reaction

**DOI:** 10.1186/s40580-022-00324-8

**Published:** 2022-07-22

**Authors:** Xun Cui, Likun Gao, Cheng-Hsin Lu, Rui Ma, Yingkui Yang, Zhiqun Lin

**Affiliations:** 1 Engineering Research Center of Nano-Geomaterials of Ministry of Education, Key Laboratory of Functional Geomaterials in China Nonmetallic Minerals Industry, Faculty of Materials Science and Chemistry, China University of Geosciences, Wuhan, 430074 China; 2 Department of Chemical and Biomolecular Engineering, National University of Singapore, 117585 Singapore, Singapore; 3 Key Laboratory of Bio-based Material Science and Technology of Ministry of Education, Northeast Forestry University, Harbin, 150040 China; 4 Key Laboratory of Catalysis and Energy Materials Chemistry of Ministry of Education, Hubei Key Laboratory of Catalysis and Materials Science, South-Central Minzu University, Wuhan, 430074 China; 5 Instrumentation Center, National Tsing Hua University, Hsinchu, 300044 Taiwan China

**Keywords:** Single-metal-atom catalyst, Electrocatalysis, Coordination structure, Oxygen reduction reaction

## Abstract

Single-metal-atom catalysts (SMACs) have garnered extensive attention for various electrocatalytic applications, owing to their maximum atom-utilization efficiency, tunable electronic structure, and remarkable catalytic performance. In particular, carbon-based SMACs exhibit optimal electrocatalytic activity for the oxygen reduction reaction (ORR) which is of paramount importance for several sustainable energy conversion and generation technologies, such as fuel cells and metal-air batteries. Despite continuous endeavors in developing various advanced carbon-based SMACs for electrocatalytic ORR, the rational regulation of coordination structure and thus the electronic structure of carbon-based SMACs remains challenging. In this review, we critically examine the role of coordination structure, including local coordination structure (i.e., metal atomic centers and the first coordination shell) and extended local coordination structure (i.e., the second and higher coordination shells), on the rational design of carbon-based SMACs for high-efficiency electrocatalytic ORR. Insights into the relevance between coordination structures and their intrinsic ORR activities are emphatically exemplified and discussed. Finally, we also propose the major challenges and future perspectives in the rational design of advanced carbon-based SMACs for electrocatalytic ORR. This review aims to emphasize the significance of coordination structure and deepen the insightful understanding of structure-performance relationships.

## Introduction

Clean and sustainable energy conversion and generation technologies play an increasingly crucial role in alleviating human beings’ dependence on conventional fossil fuels and solving global energy shortage and environmental degradation issues [1–6]. Particularly, among these technologies, fuel cells and metal-air batteries which represent the most promising devices for efficient conversion of chemical energy (e.g., hydrogen, methanol, and zinc metal) into electrical energy have garnered considerable interest over the past few decades [7–11]. Generally, the operation efficiency of these devices is primarily determined by their corresponding cathodic electrochemical process (i.e., electrocatalytic oxygen reduction reaction (ORR)) due to its relatively more sluggish kinetics in comparison with that of the anodic process [12–15]. To date, high-cost noble metal-based catalysts (e.g., commercial Pt/C) are commonly employed at the cathode/electrolyte interface in these devices to improve the catalytic efficiency of ORR, which however significantly raises the cost and greatly hampers their widespread applications [16–18]. Therefore, the development of high-efficiency noble metal-free ORR catalysts or minimizing the usage of noble metals in the catalysts is of great significance to promote the large-scale applications of these devices. To this end, massive endeavors have been devoted to developing advanced cost-effective ORR catalysts with unique features for maximizing the metal utilization, upgrading the intrinsic activity, strengthening the mass transfer, and improving the long-term durability [19–21].

Generally, the catalytic properties of a specific catalyst are highly dependent on the amount of accessible active sites and the intrinsic activity of these active sites [22, 23]. Hence, improving the number of accessible active sites within a given mass/volume and enhancing the intrinsic activity of active sites, via downsizing the geometric dimension and modulating the electronic structure, respectively, hold huge potential for the development of high-efficiency catalysts [24, 25]. Following the strategy of downsizing the geometric dimension, single-metal-atom catalysts (SMACs) have been first proposed as an emerging type of catalysts in 2011 in a pioneering study by Zhang, Li, Liu and co-workers and gradually acknowledged by the catalysis community over the last decade [26]. Thanks to the single-atom property of isolated active metal sites immobilized on the host materials, SMACs provide enormous strengths in maximizing the metal utilization and offering more uniform and well-defined active sites in comparison to the traditional heterogeneous catalysts composing of diverse kinds of poorly defined active sites [27, 28]. In the past couple of years, various SMACs have been therefore widely explored and showed encouraging electrocatalytic ORR activities [29–32]. The high intrinsic activity of isolated metal sites and the low cost of component elements in SMACs make these catalysts promising candidates to substitute traditional noble metal-based ORR catalysts [33–37]. Particularly, carbon-based SMACs exhibit the state-of-the-art electrocatalytic ORR activities, and have been accordingly recognized as an emerging type of ORR catalysts for potential practical applications in fuel cells and metal-air batteries [38–41]. In a typical carbon-based SMACs, the isolated metal sites which immobilized within a specific carbon matrix are generally coordinated with four neighboring non-metallic atoms (e.g., N and C) to form uniform and well-defined single-metal-atom active sites [42–44]. During the electrocatalytic process, the single-metal-atom sites serve as the active centers to interact with the ORR-relevant species, while the carbon matrix ensures high electronic conductivity and provides large specific surface area for accessibility of active sites and suitable pore volume distribution for mass transfer [45–47]. Although great progresses toward the synthesis and electrocatalytic ORR applications of carbon-based SMACs have been achieved recently, the key issue that how to properly design and accurately prepare the carbon-based SMACs at the atom-level to realize further enhanced intrinsic ORR activity is still not well-addressed.

**Fig. 1:**
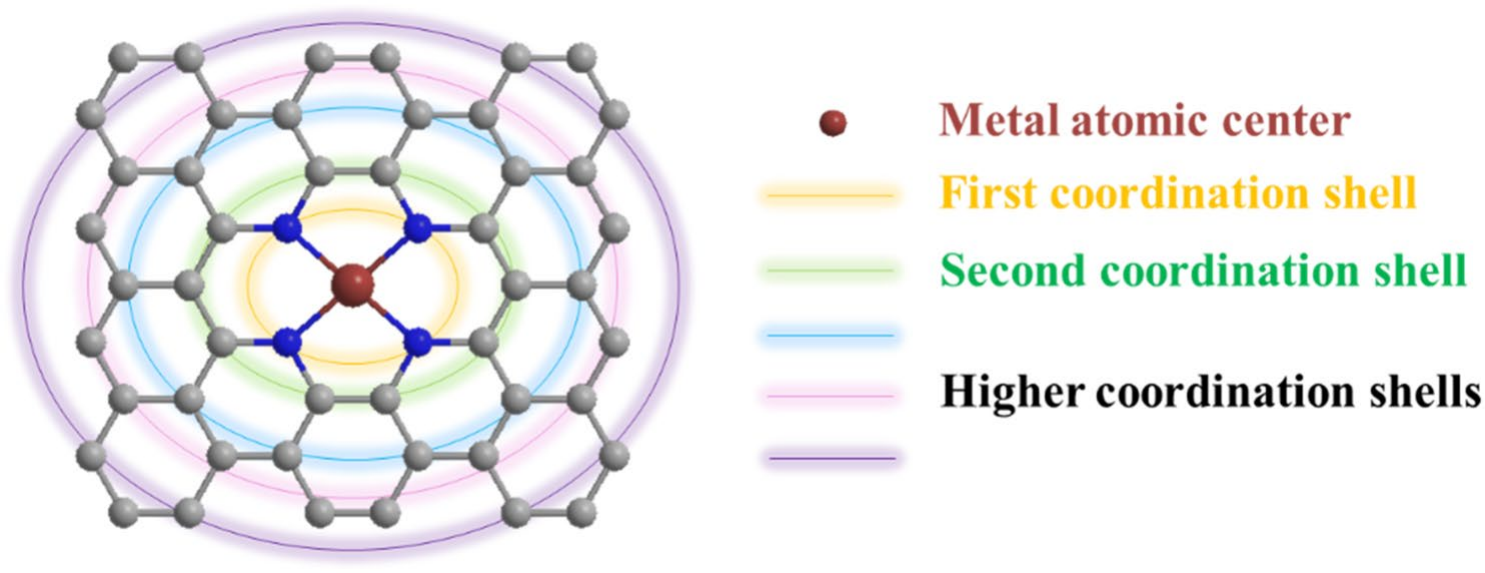
Schematic illustration of the coordination structure of carbon-based SMACs

Due to the strong electronic hybridization with coordination atoms in the vicinity of the isolated metal sites, the electronic structure of carbon-based SMACs is markedly distinct from those in nanoclusters, nanoparticles and bulk materials [48, 49]. Consequently, the coordination structure plays a decisive role in determining the intrinsic ORR activity of carbon-based SMACs [50–52]. As illustrated in Fig. 1, the coordination structure refers to local coordination structure (i.e., metal atomic center and the first coordination shell) and extended local coordination structure (i.e., the second and higher coordination shells). Specifically, since the metal atomic center is unsaturated in coordination and thus capable of directly interacting with the ORR-relevant species, therefore regulating the metal atomic center will dramatically affect the intrinsic activity of carbon-based SMACs. Furthermore, the coordination atoms in the first shell share strong electronic hybridization with the metal atomic center, tailoring these coordination atoms will also remarkably modulate the intrinsic ORR activity of carbon-based SMACs. Moreover, although the second and higher coordination shells are not directly connected with the metal atomic center, these coordination atoms could also regulate the electronic structure of carbon-based SMACs through the long-range delocalization of electron. Accordingly, the rational regulation of the coordination structure including metal atomic centers and the first, second and higher coordination shells will undoubtedly result in significantly enhanced intrinsic ORR activity of carbon-based SMACs. However, despite massive efforts in developing various advanced carbon-based SMACs, the rational regulation of the coordination structure and thus the intrinsic ORR activity remains challenging.

To date, most earlier review articles focus on the preparation strategies and the catalytic performances of SMACs [53–63]. The effect of coordination structure on the intrinsic ORR activity of carbon-based SMACs, however, has yet to be systematically examined. Therefore, a timely summary of recent advances and updated insights are highly desired to further promote the development of advanced carbon-based SMACs toward electrocatalytic ORR. In this review, we critically examine the role of coordination structure, including the metal atomic centers (Sect. 2) and the first (Sect. 3), second and higher (Sect. 4) coordination shells, on the rational design of high-efficiency carbon-based SMACs toward electrocatalytic ORR. Insights into the relevance between coordination structures and their intrinsic ORR activities are emphatically exemplified and discussed by summarizing the recent progresses. Moreover, the future challenges and opportunities in rational designing of advanced carbon-based SMACs with desired electronic properties and enhanced electrocatalytic ORR performance are also prospected. This review aims to emphasize the significance of coordination structure in carbon-based SMACs and deepen the insightful understanding of structure-activity relationships.

## Regulation of metal atomic centers

For carbon-based SMACs, various metal atomic centers that are uniformly dispersed on the carbon matrixes have been mechanically recognized as the actual active sites for electrocatalytic ORR [64–66]. Therefore, the proper selection of metal atomic center plays a pivotal role in regulating the intrinsic ORR activity of carbon-based SMACs. Generally, since the d-orbital electrons of the metal atomic centers could directly interact with the p orbitals of the O atoms in ORR-relevant species during the electrocatalytic ORR process, therefore the oxygen molecules and intermediates can be chemically adsorbed onto these isolated metal sites to initiate the subsequent multi-step electron transfer processes [67]. It has already been widely acknowledged that the lower the d-band center of the metal atomic centers, the weaker the adsorption strength of ORR-relevant species on the metal atomic centers. In fact, the weakened bonding strength of ORR-relevant species on metal atomic centers can be fundamentally ascribed to the more filling of the anti-bonding electronic states (down shifting in energy) that generated from the coupling between d orbitals of the metal atomic centers and p orbitals of the O atoms in ORR-relevant species [68].

**Fig. 2:**
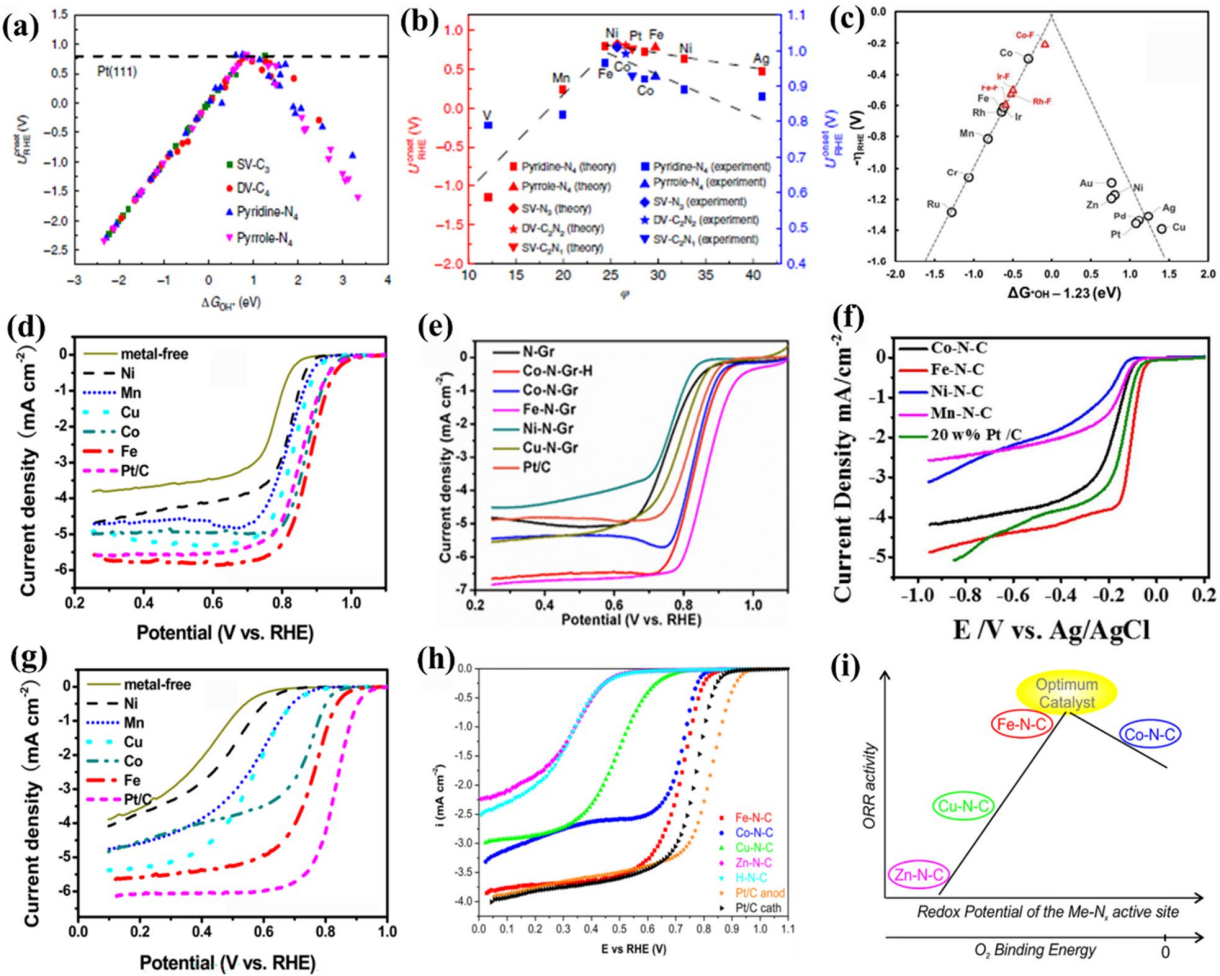
**a** ORR theoretical onset potential versus Δ*G*_OH*_ on carbon-based SMACs with different metal atomic centers. **b** Theoretical and experimental ORR onset potentials versus the descriptor *φ*. **c** The trend in the theoretical ORR overpotential of metalloporphyrin-based catalysts plotted against Δ*G*_OH*_ − 1.23 eV. **d**–**f** Linear sweep voltammetry (LSV) curves of carbon-based SMACs with different metal atomic centers in O_2_-saturated 0.1 M KOH. **g** LSV curves of carbon-based SMACs with different metal atomic centers in O_2_-saturated 0.1 M HClO_4_. **h** LSV curves of carbon-based SMACs with different metal atomic centers in O_2_-saturated 0.5 M H_2_SO_4_. **i** ORR activity versus O_2_ binding energy of carbon-based SMACs with different metal atomic centers. **a**, **b** Reproduced with permission from Ref. [75]. Copyright 2018, Nature Publishing Group. **c** Reproduced with permission from Ref. [76]. Copyright 2017, Royal Society of Chemistry. **d**, **g** Reproduced with permission from Ref. [78]. Copyright 2014, American Chemical Society. **e** Reproduced with permission from Ref. [79]. Copyright 2020, American Chemical Society. **f** Reproduced with permission from Ref. [80]. Copyright 2016, Elsevier. **h**, **i** Reproduced with permission from Ref. [81]. Copyright 2017, American Chemical Society

To date, it is still a great challenge to select the optimal metal atomic center just relied on the experimental evaluation, since some critical variables (e.g., metal-atom loadings and physicochemical properties of the carbon matrixes) that significantly affect the experimental results are hardly to be exactly controlled in actual research. Therefore, the rational choice of metal atomic center with the highest intrinsic ORR activity still remains controversial [69–74]. In this context, computational calculations have been widely employed to theoretically examine the intrinsic activities of carbon-based SMACs with a series of different metal atomic centers. As an example, Xu et al. systematically studied the intrinsic ORR activities of different carbon-based SMACs with 28 kinds of 3d, 4d, and 5d transition-metal atomic centers and demonstrated that the 3d transition-metal elements (especially Fe, Co, and their adjacent elements) possess the highest potential for high-efficiency electrocatalytic ORR [75]. Based on density functional theory (DFT) simulations, a universal volcano plot (Fig. 2a) for various carbon-based SMACs was proposed by correlating the relationships between the established activity indicator (i.e., calculated adsorption free energy for *OH (*ΔG*_OH*_)) and the theoretical onset potential (*U*_*onset*_). Notably, the Fe (i.e., Fe-pyridine-N_4_ and Fe-pyrrole-N_4_) that situated at the apex of the volcano was predicted to be the best metal atomic center of carbon-based SMACs for electrocatalytic ORR. Furthermore, a new universal descriptor (*φ*) which shows an intrinsic correlation with adsorption of ORR-relevant species and therefore with electrocatalytic activities was also identified to guide the rational selection of metal atomic centers for carbon-based SMACs (Fig. 2b). Based on the descriptor *φ*, the atomic Fe center (i.e., Fe-pyridine-N_4_ and Fe-pyrrole-N_4_) was once again recognized as the optimum metal atomic center of carbon-based SMACs for electrocatalytic ORR. In addition to the common carbon-based SMACs, it is worth noting that the descriptor *φ* can also be extended to the pyrolysis-free transition-metal-coordinated macrocyclic molecules (e.g., metallophthalocyanine- and metalloporphyrin-based covalent organic polymers) in which the metal atomic centers are also coordinated with four non-metallic atoms (e.g., N atoms in most cases) and therefore serve as active centers. However, considering the unique local chemical environment of these macrocyclic molecules, another work by Wannakao et al. computationally studied the intrinsic ORR activities of a set of metalloporphyrin-based catalysts with 14 different metal atomic centers based on DFT calculations (Fig. 2c) [76]. Intriguingly, among these metalloporphyrin-based catalysts, the atomic Co center provided the highest intrinsic ORR activity with the lowest overpotential, outperforming that of Fe and even approaching that of noble Ir center. This conclusion is further supported by the recent experimental observations by Ma et al. and Zhao et al., respectively [35, 77].

In addition to the computational evaluations, massive efforts have also been devoted into the experimental studies to explore the intrinsic ORR activity trend of different metal atomic centers in carbon-based SMACs. For instance, Peng and co-workers systematically studied the effects of different metal atomic centers (i.e., Mn, Fe, Co, Ni, and Cu) on the ORR activity of various carbon-based SMACs (Fig. 2d) [78]. They revealed that the ORR activity of the obtained carbon-based SMACs follows the sequence of Fe > Co > Cu > Mn > Ni in terms of the onset potential (*E*_*onset*_) and half-wave potential (*E*_*1/2*_). Besides, Meng et al. also prepared and investigated the ORR performance of various carbon-based SMACs with a series of high-content metal atomic centers (i.e., Fe, Co, Ni, and Cu) (Fig. 2e) [79]. They determined the electrocatalytic ORR activity trend of the prepared carbon-based SMACs as follows: Fe > Co > Cu > Ni. Similarly, another work reported by Zheng and co-authors demonstrated an active sequence of Fe > Co > Mn > Ni in their prepared carbon-based SMACs (Fig. 2f) [80]. More notably, the intrinsic activity trend of aforementioned metal atomic centers is generally valid in acidic media as well, and the only obvious difference is that the activity gap is more pronounced in acidic media in comparison with that under alkaline condition. For example, Peng et al. carried out a comparative study on the electrocatalytic ORR activity of various carbon-based SMACs with different metal atomic centers (i.e., Mn, Fe, Co, Ni, and Cu) in both acidic and alkaline media [78]. They obtained the same intrinsic ORR activity sequence of Fe > Co > Cu > Mn > Ni under both acidic and alkaline conditions (Fig. 2d,g). Similar screening research was also conducted by Osmieri and co-workers (Fig. 2 h) [81]. Through detailed analysis of experimental data, the authors proposed that the intrinsic ORR activity follows the trend of Fe > Co > Cu > Zn, which is in close relation to the redox potential of these metal-based active sites (Fig. 2i). However, despite the impressive electrocatalytic ORR performance of atomic Fe center in acidic media, Fe-related Fenton reaction usually results in rapid degradation of catalytic performance in acidic fuel cells [82]. In addition, the atomic Co center with weak Fenton effect tends to produce undesired H_2_O_2_ by-product via a two-electron pathway rather than to generate H_2_O via a desired four-electron pathway during the electrocatalytic ORR process under acidic conditions, thus possessing inferior intrinsic ORR activity [83–85]. Overall, although Fe and Co were considered to be the best choices as the optimal metal atomic centers for carbon-based SMACs under alkaline conditions, their practical applications in acidic fuel cells remain highly challenging. Other strategies to further optimize the electronic structures of carbon-based SMACs and thus tailoring their intrinsic activity should be considered specifically.

## Regulation of the first coordination shell

In carbon-based SMACs, the coordination atoms in the first shell refer to the atoms directly bonded to the metal atomic centers via d-p σ-bonds and possible π-back-donation interactions. In this context, these coordination atoms share strong electronic hybridization with the metal atomic centers. In fact, the coordinated metal atomic centers can be recognized as typical chelates in terms of coordination chemistry. Generally, according to the crystal field theory, the d-orbitals of a four-coordinated metal atomic center with a square-planar structure (D_4 h_ symmetry) are split into five partial d-orbitals in the following order of d_xz_ and d_yz_, ${\text d}_{{\rm z}^{2}}$, d_xy_, and ${\text d}_{{\rm x}^{2}-{\rm y}^{2}}$, respectively [86–88]. Based on the ligand field theory, when the orbitals of ligands with proper symmetry approach the split d-orbitals, the σ and π bonding and anti-bonding molecular orbitals can be produced [86]. Therefore, the crystal field of the coordinated metal atomic center and the splitting and binding energies of the metal atomic center are dominantly determined by the surrounding ligands (i.e., coordination atoms). Since the atomic architecture of first shell is defined by the number and type of coordination atoms, tailoring these properties may result in unique electronic structures (d orbitals) of metal atomic centers to further enhance the intrinsic ORR activity of carbon-based SMACs. In this section, we highlight recent advances in regulating the first coordination shell of various carbon-based SMACs for high-performance electrocatalytic ORR.

### Coordination number

**Fig. 3:**
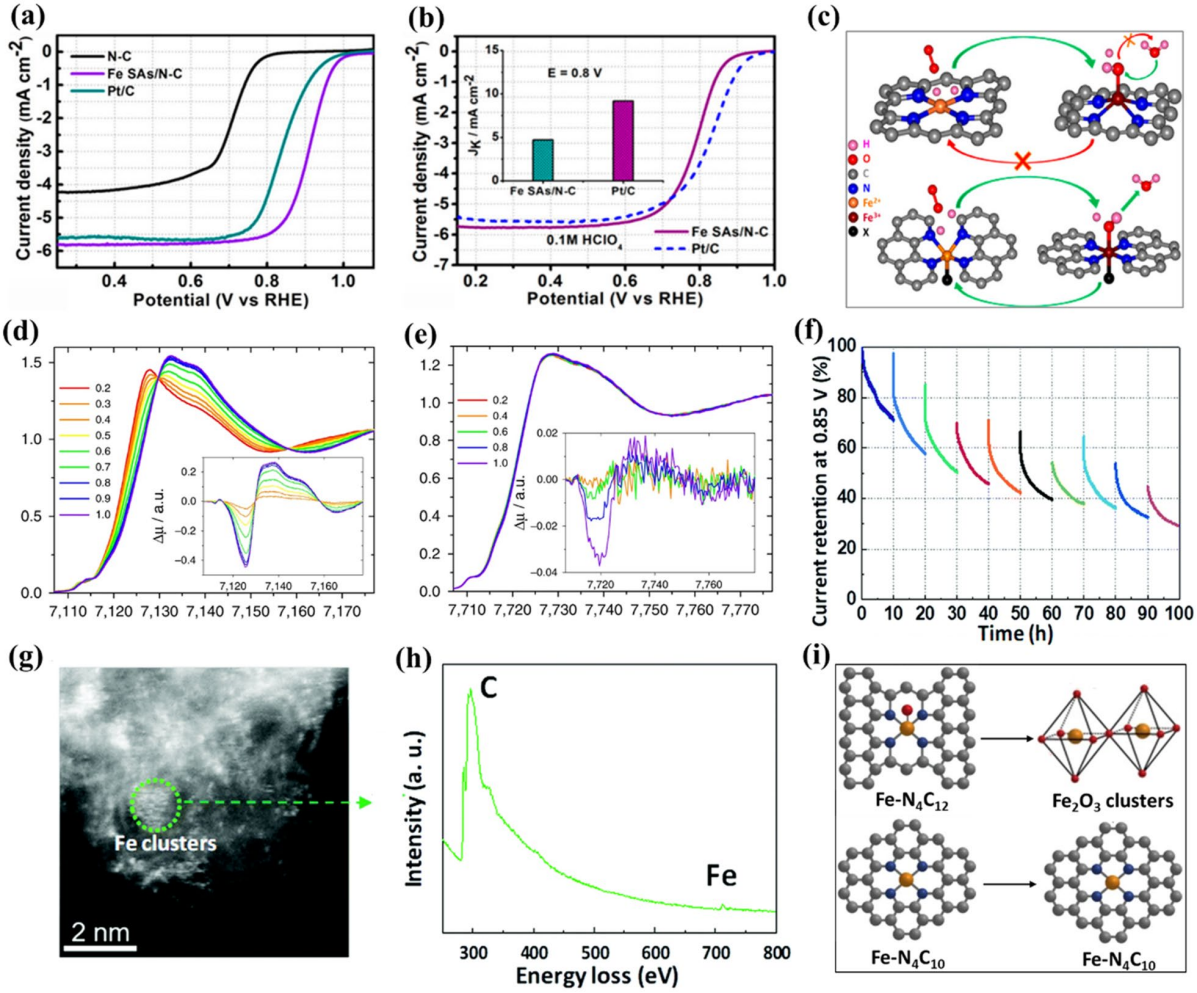
**a** LSV curves of N-C, Fe SAs/N-C and Pt/C catalysts in O_2_-saturated 0.1 M KOH. **b** LSV curves of Fe SAs/N-C and Pt/C catalysts in O_2_-saturated 0.1 M HClO_4_. **c** Schematic illustration of Fe-N switching behaviour of Fe-N_4_-C_x_ models with/without axially bound O(H)_ads_. *Operando* X-ray absorption near-edge structure (XANES) spectra of **d** Fe-N_4_ and **e** Co-N_4_ catalysts (insets: differential Δµ XANES spectra). **f** Durability testing of Fe-N_4_ catalyst at 0.85 V for 100 h. **g** High angle annular dark field scanning transmission electron microscopy (HAADF-STEM) image and **h** electron energy loss spectroscopy (EELS) analysis of Fe-N_4_ catalyst after the durability testing. **i** Schematic illustration of coordination or structural changes of Fe-N_4_C_12_ and Fe-N_4_C_10_ sites, respectively. **a**, **b** Reproduced with permission from Ref. [89]. Copyright 2019, American Chemical Society. **c** Reproduced with permission from Ref. [92]. Copyright 2015, American Chemical Society. **d**, **e** Reproduced with permission from Ref. [93]. Copyright 2017, Nature Publishing Group. **f**–**h** Reproduced with permission from Ref. [42]. Copyright 2019, Royal Society of Chemistry. **i** Reproduced with permission from Ref. [94]. Copyright 2021, Nature Publishing Group

To date, numerous carbon-based SMACs with M-N_x_ (x represents coordination number) active centers have been verified to display superior electrocatalytic ORR activity to their nanoclusters, nanoparticles and bulk materials counterparts. In most of these reported carbon-based SMACs, the common coordination number of metal atomic centers is four (i.e., M-N_4_ with a square-planar structure) as a result of the valence states and electronic structures of central metal atoms. For example, Yang et al. reported the synthesis of a Fe-N_4_ sites decorated porous N-doped carbon (Fe SAs/N-C) for electrocatalytic ORR via a molecules-confined pyrolysis approach [89]. The Fe-N_4_ sites which prefer to have a high O coverage and possess high four-electron pathway selectivity have been demonstrated to endow excellent electrocatalytic ORR activity in both acidic media (*E*_*1/2*_ of 0.798 V) and alkaline (*E*_*1/2*_ of 0.91 V) solution (Fig. 3a,b). Similarly, Zhang et al. employed a modular strategy to incorporate Co-N_4_ sites into a multichannel carbon matrix (Co@MCM) for efficient electrocatalytic ORR [90]. Benefiting from the advantageous contribution of atomic Co centers to the charge density distribution, the hydrogenation of *O_2_ species on Co-N_4_ sites was greatly accelerated. As a result, the Co@MCM exhibited excellent activity for electrocatalytic ORR in O_2_-saturated 0.1 M KOH solution (*E*_*onset*_ of 0.95 V and *E*_*1/2*_ of 0.86 V). The authors also demonstrated the considerable electrocatalytic ORR performance of Co@MCM under acidic conditions. Besides, Jung et al. recently synthesized a Co-N_4_ sites decorated single-wall carbon nanohorns catalyst via an ammonia annealing process for efficient electrocatalytic ORR [91]. By employing DFT calculations, they attributed the high intrinsic electrocatalytic ORR activity of the Co-N_4_ catalyst to a ligand-push effect of water molecules to the Co-N_4_ sites in the axial direction. Based on the crystal field theory, they proposed that the approaching of unshared electron pair of water molecule to Co-N_4_ site in the axial direction could strongly destabilize the ${\text d}_{{\rm z}^{2}}$ level with respect to d_xz_ and d_yz_, benefitting for the axial bonding between metal Co center and the ORR-relevant species. As a result, the Co-N_4_ catalyst exhibited a much higher ORR kinetic activity of 60.16 mA cm^− 2^ at 0.8 V in O_2_-saturated 0.1 M KOH electrolyte solution, which is 7.3 times higher than that of commercial Pt/C catalyst (8.24 mA cm^− 2^).

In addition to aforementioned results based on static descriptions, it is worth noting that the dynamic evolution of M-N_4_ catalytic center during the electrocatalytic ORR has been captured very recently. As a typical example, by employing *operando* X-ray absorption near-edge spectroscopy (XANES), Jia et al. observed that the Fe center in Fe-N_4_ site dynamically moving toward or away from the N_4_-plane during the electrocatalytic ORR (Fig. 3c) [92]. They demonstrated that the Fe^2+^ center is protruded out of the N_4_-plane in non-planar Fe^2+^-N_4_-C at a lower potential. While upon anodic polarization over the Fe^2+^/Fe^3+^ redox potential, besides the oxidation of Fe^2+^ to Fe^3+^, an additional oxygen atom will coordinate with the Fe^3+^ center, thereby resulting in a planar (H)O-Fe^3+^-N_4_-C structure (Fig. 3c bottom). Notably, the dynamic evolution of non-planar Fe^2+^-N_4_-C to planar (H)O-Fe^3+^-N_4_-C was well-correlated with the ORR performance, revealing that the distorted Fe^2+^-N_4_-C sites are the main active sites. Similarly, Zitolo et al. recently also reported that the Fe-N_4_ sites experience dynamic evolution (i.e., structural and electronic-state changes) with the applied potential during the electrocatalytic ORR process, while no obvious change was observed in the case of Co-N_4_ sites from 0 to 1.0 V, indicating different electrocatalytic ORR mechanisms for each M-N_4_ structure (Fig. 3d, e) [93]. It is notable that the energies of the d_xz_, d_yz_, and ${\text d}_{{\rm z}^{2}}$ orbitals change significantly when the metal atomic center moves toward or away from the N_4_-plane, while those of d_xy_ and ${\text d}_{{\rm x}^{2}-{\rm y}^{2}}$ change slightly, finally resulting in the state change of d orbitals [87]. Moreover, recent reports have also demonstrated that the M-N_4_ catalytic centers could degrade dynamically during the electrocatalytic ORR process. For instance, Zhang et al. observed a continuous degradation of ORR performance for a Fe-N_4_ catalyst with a long-term constant-potential testing at 0.85 V for 100 h, which can be ascribed to the dynamic demetalation and formation of Fe clusters (Fig. 3f−h) [42]. Moreover, Li et al. discovered that the high-spin Fe-N_4_C_12_ sites could quickly transform to ferric oxides after a long-term operation (50 h) of electrocatalytic ORR, while the low- or intermediate-spin Fe-N_4_C_10_ sites remained stable (Fig. 3i) [94]. These findings highlight the significance of the dynamic evolution of M-N_4_ catalytic centers during the electrocatalytic ORR process. However, it is noteworthy that the dynamic response mechanisms of catalytic centers to the environment and catalytic functionality as well as the structure-performance relations of these as-evolved active sites are still difficult to be established experimentally. Clearly, further theoretical simulation is still greatly needed.

**Fig. 4:**
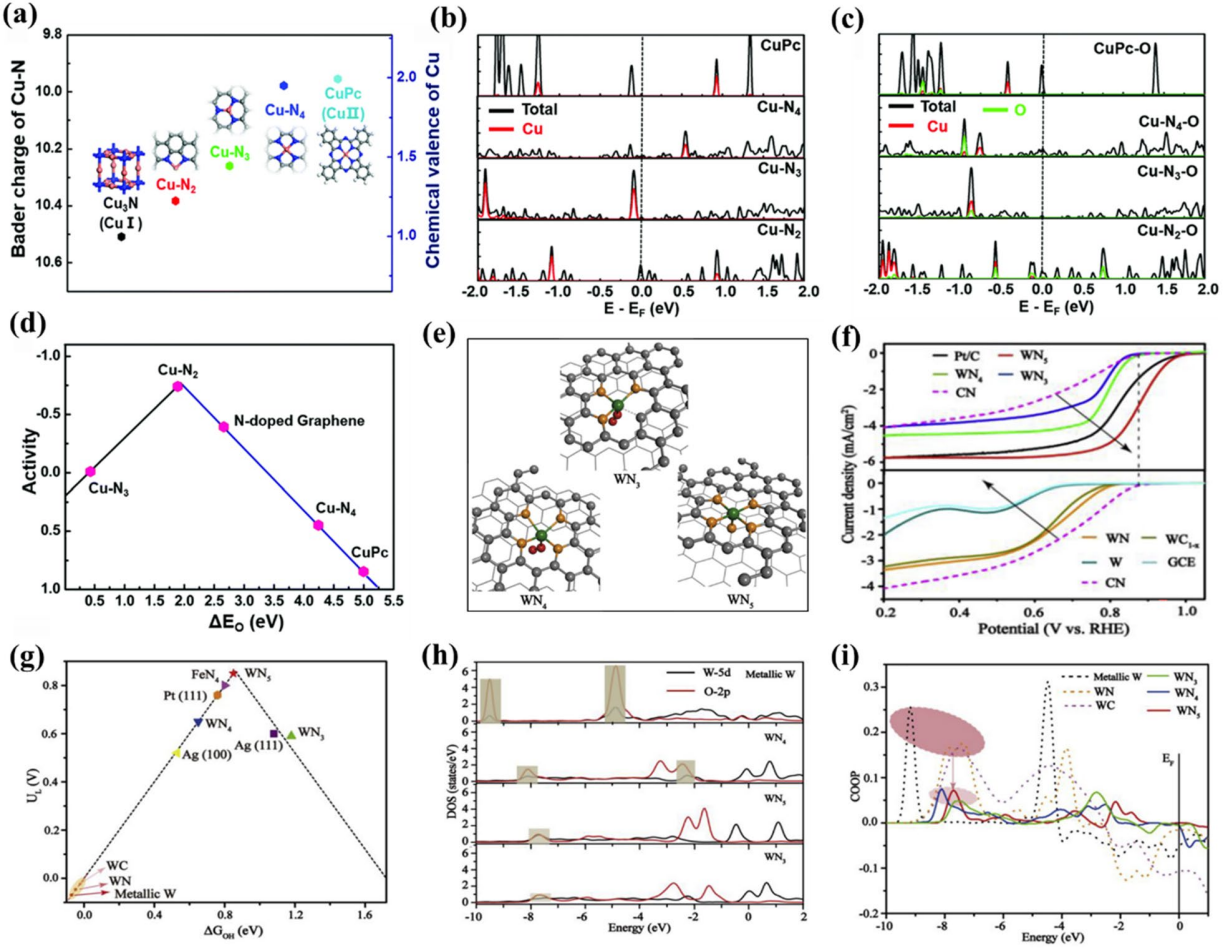
**a** Bader charge and chemical valence of Cu centers for different structures: Cu_3_N bulk, Cu-N_2_, Cu-N_3_, Cu-N_4_ and CuPc molecule. **b** Total density of states (DOS) (black) for the CuPc, Cu-N_4_, Cu-N_3_ and Cu-N_2_, and projected DOS of Cu atoms (red) from them. **c** Total DOS (black) for one O adsorbed CuPc, Cu-N_4_, Cu-N_3_ and Cu-N_4_, and projected DOS of Cu atoms (red) and O atoms (green) from them. **d** Volcano plot of the relationship between ORR activity and Δ*E*_O_ for Cu-N_2_, Cu-N_3_, Cu-N_4_, CuPc and N-doped graphene. **e** Schematic models of W-N_3_, W-N_4_ and W-N_5_, respectively. W (deep green), N (orange), O (red), and C (grey). **f** LSV curves of W-N_3_, W-N_4_, W-N_5_, CN, and Pt/C catalysts in O_2_-saturated 0.1 M KOH. (g) Volcano plot of the relationship between the limiting potential (*U*_*L*_) and Δ*G*_OH_. **h** Local density of the states of W and O for *OH absorbed on metallic W, W-N_3_, W-N_4_ and W-N_5_. **i** Crystal orbital overlap population (COOP) analysis results for *OH absorbed on W-N_3_, W-N_4_ and W-N_5_. **a**–**d** Reproduced with permission from Ref. [95]. Copyright 2016, Royal Society of Chemistry. (e-i) Reproduced with permission from Ref. [65]. Copyright 2019, Elsevier

**Fig. 5:**
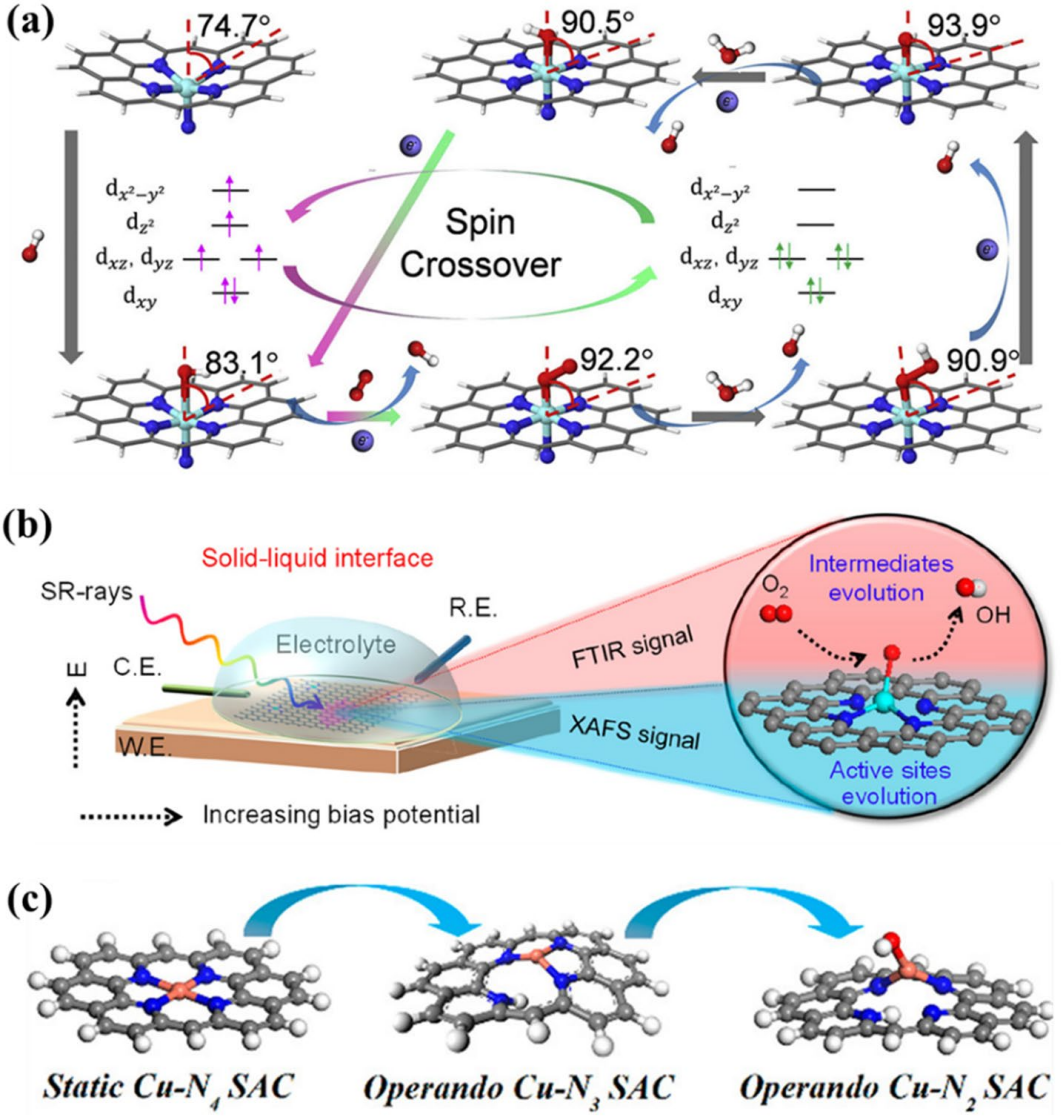
**a** Structural and dynamics of Fe-N_5_C_10_ site in electrocatalytic ORR. **b** Schematic illustration of the *operando* synchrotron characterization (C.E., counter electrode; R.E., reference electrode; W.E., working electrode) and the dynamic evolution of Ni-N_4_ site. **c** Schematic illustration of the dynamic evolution of Cu-N_4_ site. **a** Reproduced with permission from Ref. [99]. Copyright 2020, Elsevier. **b** Reproduced with permission from Ref. [100]. Copyright 2020, American Chemical Society. **c** Reproduced with permission from Ref. [101]. Copyright 2021, American Chemical Society

The coordination number of metal atomic centers has also been widely explored and validated to have remarkable influence on the electronic properties and electrocatalytic ORR activities of carbon-based SMACs. For instance, Wu et al. reported the preparation of graphene supported Cu-N_2_ sites via pyrolysis of copper phthalocyanine (CuPc) and dicyandiamide for efficient electrocatalytic ORR [95]. Compared with Cu-N_4_ sites and CuPc molecules with a valence state of ≈ 2.0 (Fig. 4a), the authors demonstrated that the Cu-N_2_ sites with a valence state of ≈ 1.2 were more conducive to *O_2_ adsorption during the electrocatalytic ORR due to their stronger chemical interactions. Further projected density of states (DOS) analysis (Fig. 4b, c) revealed that the presence of some new hybridized electronic states above Fermi level resulted in less occupation of anti-bonding states of O-Cu-N_2_ in comparison to O-Cu-N_4_ and O-CuPc. As a result, the Cu-N_2_ sites possessed an intermediate binding strength with the oxygen species (Fig. 4d), leading to both good adsorption and desorption abilities during the electrocatalytic ORR, thus improving the overall ORR performance. Furthermore, Yin et al. proposed that the stronger interaction with H_2_O_2_ intermediate on Co-N_2_ compared with that on Co-N_4_ sites could facilitates the four-electron pathway of electrocatalytic ORR process [96]. More importantly, Chen and co-workers reported that the W-N_x_ sites decorated N-doped carbon nanosheets with precisely adjustable coordination numbers (Fig. 4e) could serve as remarkable catalysts toward electrocatalytic ORR under both alkaline and acidic conditions, in sharp contrast with other ORR-inert 5d W-based catalysts [65]. The experimental results clearly demonstrated that the W-N_5_ sites display superior electrocatalytic ORR performance (*E*_*onset*_ of 1.01 V and *E*_*1/2*_ of 0.88 V) compared with that of W-N_3_ and W-N_4_ sites (Fig. 4f). Based on systematic DFT calculations, the authors constructed an activity volcano plot (Fig. 4g) by correlating the calculated free energy of the adsorbed *OH ((*ΔG*_*OH*_)) with the potential limiting step (i.e., *U*_*L*_). Clearly, both W-N_3_ and W-N_4_ sites bound *OH either too strong (W-N_4_) or too weak (W-N_3_), making them unsuitable for high-efficiency electrocatalytic ORR. The DOS (Fig. 4h) and crystal orbital overlap population (COOP) (Fig. 4i) analysis further revealed that the W-N_5_ sites offer moderate adsorption strength towards *OH, which plays a decisive role for W-N_5_ sites in electrocatalytic ORR. In addition, Huang and co-workers also found that the Fe-N_5_ sites displayed better intrinsic ORR activity than Fe-N_4_ sites based on their experimental studies [97]. The computational simulations revealed that the electron push effect of axial-coordinated N in Fe-N_5_ sites during adsorption could fill the antibonding π* orbital of O_2_ molecule, thus resulting in weakened O-O bond and facilitating the electrocatalytic ORR process. Lai and co-workers also obtained similar results that the five-coordinated Fe-N_5_ sites could markedly upgrade the electrocatalytic ORR activity by lowering the energy barrier and reducing the adsorption energy of *OH intermediate [98]. Therefore, for metal atomic centers that are dominated by the d-band, if the adsorption toward ORR-relevant species is strong, raising the coordination number generally could weaken the adsorption and therefore facilitate the electrocatalytic ORR process, and vice versa. In addition to the static descriptions, Li et al. recently employed *operando* ^57^Fe Mossbauer spectroscopy to explore the potential-relevant dynamic evolution of single-Fe-atom catalyst [99]. They reported that the electronic state of single-atom Fe^2+^ transformed from high-spin to low-spin upon the formation of O_2_^−^-Fe^2+^-N_5_ near the *E*_*onset*_ (0.9 V), while a contrary conversion from low-spin to high-spin was discovered upon the formation of O_2_^−^-Fe-N_4_ at lower potentials of 0.7 and 0.5 V. Notably, the adsorption-induced distortion of the coordination configuration directly results in the refinement of the ligand field around the metal atomic Fe centers, which is a prerequisite for attaining the strong interaction with ORR-relevant species. On the basis of these observations, they proposed a spin-crossover-involved ORR mechanism in which the high-spin Fe-N_5_C_10_ sites were recognized as the most active sites toward electrocatalytic ORR (Fig. 5a). This work exemplified that the external stimuli (i.e., applied potential) may lead to dynamic evolution of active site and make the spin states of the active site interconvertible.

In addition, recent studies have demonstrated that dynamic evolution of catalytic centers during electrocatalytic ORR significantly affects the coordination number of metal atomic centers. For example, by combining multiple *operando* synchrotron spectroscopies, Su et al. observed that Ni centers in porphyrin-like Ni-N_4_ sites tend to be dynamically released from the N-doped carbon matrix, resulting in the formation of near-free and isolated-zigzag Ni^(2−δ)+^N_2_ active sites during electrocatalytic ORR process (Fig. 5b) [100]. The as-evolved Ni^(2−δ)+^N_2_ active sites were verified to promote the adsorption and dissociation of O_2_ molecule into a crucial *O intermediate, thereby boosting the electrocatalytic ORR performance. Similarly, Yang et al. identified the dynamic evolution of Cu-N_4_ to Cu-N_3_ and further to HO-Cu-N_2_ during the electrocatalytic ORR process, which concurrently occurs with the reduction of Cu^2+^ to Cu^+^ driven by the applied potential (Fig. 5c) [101]. The increase in Cu^+^/Cu^2+^ ratio with the applied potential reveals that the as-evolved low-coordinated Cu^+^-N_3_ sites are the real active sites for electrocatalytic ORR. Clearly, the planar M-N_4_ configurations in both cases were demolished during the electrocatalytic ORR process by partial removal of the coordinated N atoms, leading to the formation of new low-coordinated active sites. These results offer new perspectives for electrocatalytic ORR mechanisms of carbon-based M-N_4_ catalysts.

### Coordination atom

**Fig. 6:**
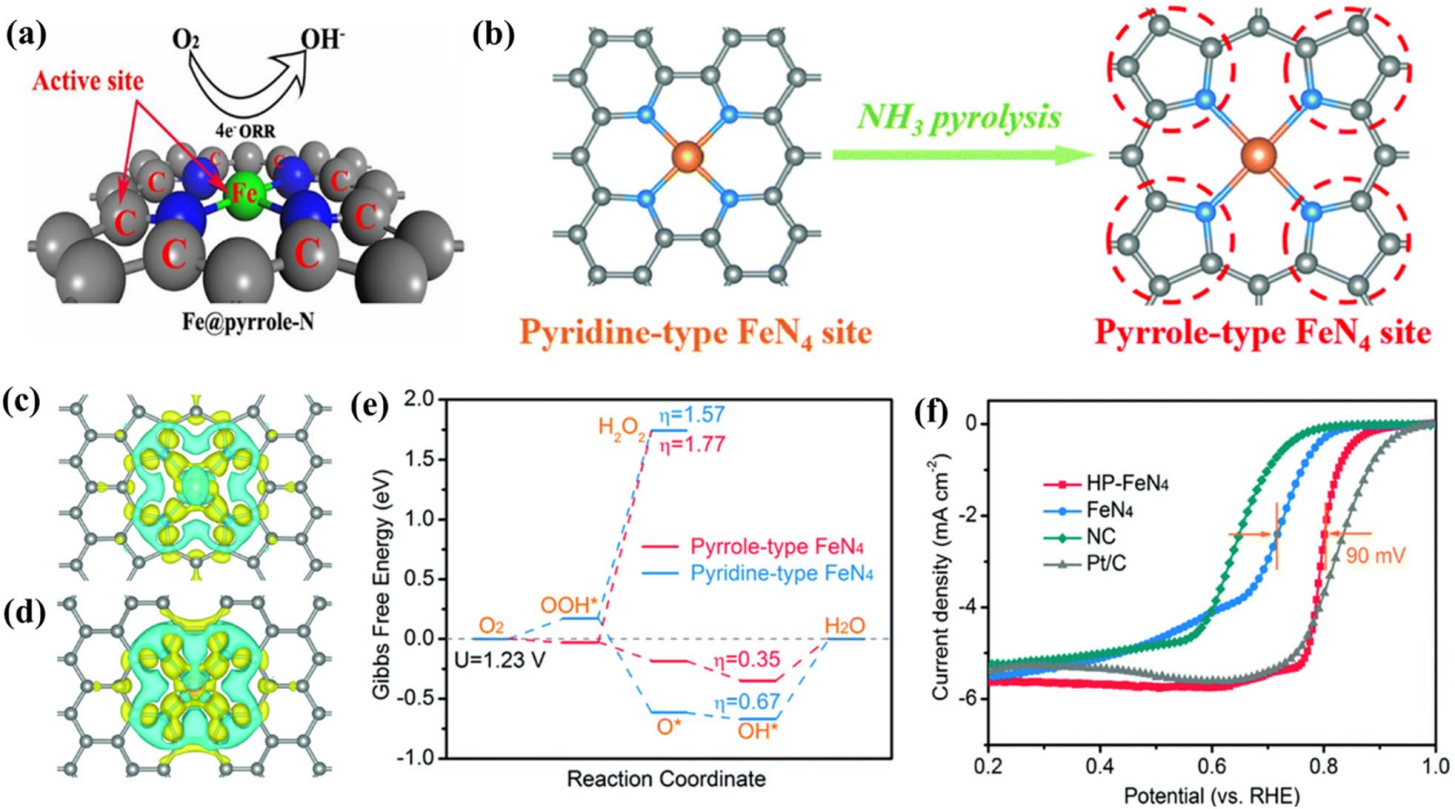
**a** Structural model of Fe-pyrrolic-N_4_ site (Fe@pyrrolic-N) for ORR, where both Fe atom and eight-carbon atoms next to pyrrolic-N are the active centers. **b** Preparation process of high-purity Fe-pyrrolic-N_4_ structure. The balls in grey, blue and orange represent C, N and Fe atoms, respectively. **c**, **d** Calculated charge density difference of **c** Fe-pyrrolic-N_4_ and **d** Fe-pyridinic-N_4_. Yellow and blue regions represent electron accumulation and electron depletion, respectively. **e** Free energy diagram of ORR on Fe-pyrrolic-N_4_ and Fe-pyridinic-N_4_ sites. **f** LSV curves of Fe-pyrrolic-N_4_ (HP-FeN_4_), Fe-pyridinic-N_4_ (FeN_4_) and NC catalysts in O_2_-saturated 0.5 M H_2_SO_4_ and 20% Pt/C in 0.1 M HClO_4_. **a** Reproduced with permission from Ref. [102]. Copyright 2018, National Academy of Sciences. **b**–**f** Reproduced with permission from Ref. [103]. Copyright 2020, Royal Society of Chemistry

Apart from the coordination number of metal atomic centers, the type of coordination atoms also can be regulated to tailor the electrocatalytic ORR activity of carbon-based SMACs. Particularly, for the most common carbon-based SMACs with M-N_4_ sites as active centers, two types of typical coordination N atoms (i.e., pyridinic-N and pyrrolic-N) have been previously reported. For instance, Yang et al. comparatively evaluated the intrinsic electrocatalytic ORR performance of Fe-pyrrolic-N_4_ and Fe-pyridinic-N_4_ sites by combining experimental studies with computational calculations [102]. The Fe-pyrrolic-N_4_ sites were verified to be more active for catalyzing oxygen reduction than Fe-pyridinic-N_4_ sites as the Fe central atoms in Fe-pyrrolic-N_4_ could activate the eight carbon atoms next to the four pyrrolic-N atoms to serve as extra active centers for the electrocatalytic ORR (Fig. 6a). The synergistic effects between Fe central atoms and these activated carbon atoms resulted in significantly improved electrocatalytic ORR activity of Fe-pyrrolic-N_4_ sites. In sharp contrast, no such synergistic effects exist in Fe-pyridinic-N_4_ sites, giving rise to lower electrocatalytic ORR activity. Similarly, Zhang et al. recently reported the superior electrocatalytic ORR activity of Fe-pyrrolic-N_4_ sites via constructing high-purity Fe-pyrrolic-N_4_ sites through an ammonia-assisted pyrolysis strategy (Fig. 6b) [103]. The DFT calculations demonstrated that the pyrrolic-N atoms in Fe-pyrrolic-N_4_ donated less electrons to the Fe central atom in comparison with that of Fe-pyridinic-N_4_, thereby leading to a more positive valence state of Fe central atom in Fe-pyrrolic-N_4_ sites (Fig. 6c,d). Furthermore, the preferred O_2_ adsorption energy, reduced energy barrier of the rate-limiting step (*OH to H_2_O), and higher four-electron pathway selectivity rendered a better intrinsic ORR activity of Fe-pyrrolic-N_4_ sites (Fig. 6e). As a result, the Fe-pyrrolic-N_4_ sites exhibited markedly improved *E*_*onset*_ (0.95 V) and *E*_*1/2*_ (0.80 V) compared with those of Fe-pyridinic-N_4_ sites (0.86 V and 0.71 V, respectively) in acidic media (Fig. 6f). In addition, it has also been demonstrated that the micropores-hosted Fe-pyridinic-N_4_ sites could enhance the electrocatalytic ORR activity [104]. The improved electrocatalytic ORR activity can be mechanistically attributed to the more readily breaking of O-O bond in *OOH adsorbed on micropores-hosted Fe-pyridinic-N_4_ sites which resulted in lowered activation energy.

In addition to N, the metal atomic centers in carbon-based SMACs can also be coordinated with other non-metallic atoms (e.g., C, S, P, B, O, etc.). It has been validated that these coordination atoms are capable of regulating the electronic structures of the metal atomic centers. For carbon-based SMACs, atomically dispersed metal centers often connected directly to the carbon host materials to form M-C_x_ chemical coordination. As an example, Yin et al. reported that the ratio of coordination atoms (i.e., N and C) for metal atomic centers in carbon-based SMACs can be readily modulated by controlling the pyrolysis temperature of precursors [96]. Two different atomic Co centers that coordinated with four N atoms (Co-N_4_) and two N atoms (Co-N_2_C_2_), were obtained in this work at pyrolysis temperature of 800 ℃ and 900 ℃, respectively. Compared with the common Co-N_4_, the Co-N_2_C_2_ sites exhibited stronger interactions with H_2_O_2_ intermediate, resulting in significantly improved electrocatalytic ORR activity with high four-electron pathway selectivity. In addition, Liu et al. demonstrated that the main-group Mg central atom coordinated with two N atoms (Mg-N_2_C_2_) possess the near-optimal adsorption capacity with ORR-relevant species as a result of the rise of p-band center position in comparison to other coordination environments (i.e., Mg-N_1_C_2_, Mg-N_3_, and Mg-N_4_) [105]. Accordingly, the Mg-N_2_C_2_ exhibited a remarkable electrocatalytic ORR performance with *E*_*1/2*_ of 910 mV and *E*_*onset*_ of 1.03 V in alkaline media.

**Fig. 7:**
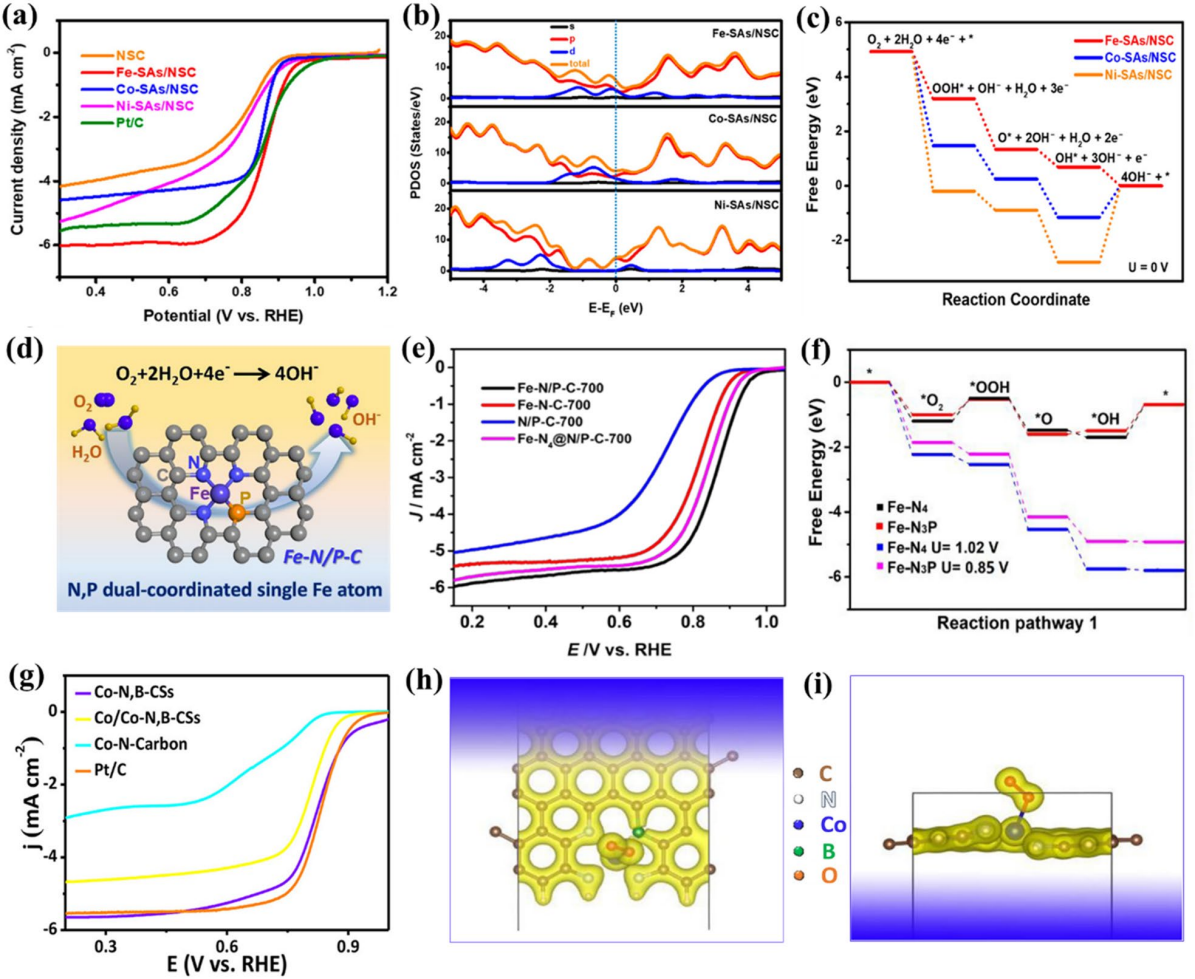
**a** LSV curves of Fe-SAs/NSC (Fe-N_4_S_2_), Co-SAs/NSC (Co-N_3_S_1_), Ni-SAs/NSC (Ni-N_3_S_1_) and reference samples in O_2_-saturated 0.1 M KOH solution. **b** The calculated partial density of states (PDOS) of Fe-SAs/NSC (Fe-N_4_S_2_), Co-SAs/NSC (Co-N_3_S_1_), and Ni-SAs/NSC (Ni-N_3_S_1_). **c** Free energy diagrams of ORR on Fe-SAs/NSC (Fe-N_4_S_2_), Co-SAs/NSC (Co-N_3_S_1_), and Ni-SAs/NSC (Ni-N_3_S_1_). **d** Structural model of Fe-N_3_P_1_ site for ORR. **e** LSV curves of Fe-N/P-C-700 (Fe-N_3_P_1_), Fe-N-C-700 (Fe-N_4_), N/P-C-700, and Fe-N_4_@N/P-C-700 (Fe-N_4_) in O_2_-saturated 0.1 M KOH solution. **f** Free energy diagrams of ORR on Fe-N_4_ and Fe-N_3_P_1_ sites. **g** LSV curves of Co-N,B-CSs (Co-N_3_B_1_), Co/Co-N,B-CSs, Co-N-Carbon (Co-N_4_), and Pt/C catalysts in O_2_-saturated 0.1 M KOH solution. **h**, **i** Optimized geometry of the corresponding O_2_ adsorption configuration on Co-N,B-CSs (Co-N_3_B_1_). **a**–**c** Reproduced with permission from Ref. [106]. Copyright 2019, American Chemical Society. **d**–**f** Reproduced with permission from Ref. [110]. Copyright 2020, American Chemical Society. **g**-**i** Reproduced with permission from Ref. [112]. Copyright 2018, American Chemical Society

Beyond C atom, S atom has also been proposed as coordination atoms to enhance the intrinsic ORR activity of metal atomic centers in carbon-based SMACs. For instance, Zhang et al. recently prepared a series of carbon-based SMACs with well-dispersed Fe-N_4_S_2_, Co-N_3_S_1_, and Ni-N_3_S_1_ sites, respectively, as ORR catalysts to explore the structure-performance correlations [106]. Electrochemical measurements revealed that the Fe-N_4_S_2_ sites have the best ORR performance with *E*_*1/2*_ of 0.87 V and *E*_*onset*_ of 1.00 V under alkaline conditions, significantly larger than those of Co-N_3_S_1_ (0.86 and 0.95 V, respectively) and Ni-N_3_S_1_ (0.82 and 0.96 V, respectively) sites (Fig. 7a). DFT calculations demonstrated that the Fe-N_4_S_2_ sites possess the highest density states near the Fermi level in comparison to Co-N_3_S_1_ and Ni-N_3_S_1_ sites, indicating greatly enhanced electron transfer for Fe-N_4_S_2_ sites (Fig. 7b). As a result, the Fe-N_4_S_2_ sites displayed the lowest energy barriers for electrocatalytic ORR (Fig. 7c). Similarly, another work reported by Zhang and co-authors also demonstrated the superior electrocatalytic ORR activity of Fe-N_3_S_1_ sites compared to Fe-N_4_ active sites [107]. Furthermore, Shang et al. found that after the incorporation of S coordination atoms, more occupying of electrons occurred in the ${\text d}_{{\rm x}^{2}-{\rm y}^{2}}$ orbital of Cu atoms for the Cu-N_3_S_1_ sites compared to Cu-N_4_ sites [108]. The electrons in the ${\text d}_{{\rm x}^{2}-{\rm y}^{2}}$ orbital could further interact with the p orbital of O atoms in ORR-relevant species and then generate additional π bonds, leading to strengthening of the bonding of oxygen intermediates and thus boosting the electrocatalytic ORR performance. In addition, Chen and co-workers’ work also highlights the pivotal role of S coordination atoms in Cu-N_3_S_1_ sites on the activation of the electron transfer around Cu-N_3_S_1_ and the enhancement of the interactions with ORR-relevant species during the electrocatalytic ORR process [109].

Apart from S atom, the positive effects of P coordination atom on the electrocatalytic ORR activity has also been demonstrated recently. As an example, Yuan and co-workers reported the synthesis and electrocatalytic ORR performance of N and P dual-coordinated Fe sites (Fe-N_3_P_1_) anchored on carbon matrix (Fig. 7d) [110]. Based on systemic electrochemical studies, the Fe-N_3_P_1_ sites exhibited superior electrocatalytic ORR activity with *E*_*1/2*_ of 0.867 V and *E*_*onset*_ of 0.941 V under alkaline conditions compared to the Fe-N_4_ sites (Fig. 7e). DFT calculations demonstrated that the O_2_ molecules can be readily adsorbed on the Fe-N_3_P_1_ sites, and more importantly the Fe-N_3_P_1_ sites were verified to be less endothermic (0.85 eV) than that of the Fe-N_4_ sites (1.02 eV) during the rate-determining step (*OH to OH^−^), indicating that Fe-N_3_P_1_ sites are more thermodynamically favourable for electrocatalytic ORR (Fig. 7f). Besides, Wei et al. also figured out that more electrons could accumulate on oxygen when it adsorbed on the Co-N_2_P_2_ sites compared to the Co-N_4_ sites, suggesting more efficient bonding with oxygen, which is conducive to the electrochemical reduction of O_2_ [111].

In addition, the intrinsic ORR activity of the Co-N_4_ sites differs markedly from Co-N_3_B_1_ sites when one adjacent N in the first shell is replaced by B atom [112]. As reported by Guo et al., the Co-N_3_B_1_ sites (Co-N,B-CSs) exhibited an apparent Pt/C-like electrocatalytic ORR activity with *E*_*1/2*_ of 0.83 V and limiting current density of 5.66 mA cm^− 2^, significantly outperforming that of the Co-N_4_ sites (Co-N-Carbon; 0.64 V and 3.13 mA cm^− 2^, respectively) (Fig. 7 g). DFT calculations revealed that the incorporation of B atom into the first coordination shell could cause the unbalanced charge distribution around the metal atomic center, hence positively polarizing the Co-N_3_B_1_ sites, which is beneficial to the adsorption of ORR-relevant species and accelerates the four-electron pathway kinetics during electrocatalytic ORR (Fig. 7 h,i). Moreover, Pan et al. systematically investigated the relationship between the coordination field and two-electron ORR activity of M-N_x_B_y_ based on DFT calculations [113]. They demonstrated that the Ni-containing graphene with N, B and Ni in a hexatomic ring (Ni-N_2_B_2_-h) exhibited the highest activity with an ultralow overpotential of 0.12 V. The excellent two-electron ORR activity of Ni-N_2_B_2_-h can be attributed to the appropriate ligand field of Ni ions which could mediate the coordination interaction of the ligand and back-donating interaction of the metal Ni center, resulting in partial O_2_ reduction. Moreover, carbon-supported N and O dual-coordinated Mn sites (Mn-N_x_O_y_) also have been proposed as efficient ORR catalysts [114]. The electrochemical measurements revealed that the Mn-N_x_O_y_ sites displayed higher electrocatalytic ORR activity than that of the Mn-N_4_ sites. Further DFT calculations on Mn-N_1_O_3_, Mn-N_2_O_2_ and Mn-N_3_O_1_ sites suggested that the down shifting of d-band center position and adjacent position of first peak relative to Fermi level in Mn-N_3_O_1_ sites renders the lowest energy barrier and fastest electrocatalytic ORR kinetics. All above recent advances demonstrate that rational regulation of non-metallic coordination atoms with different electronegativity in the first shell of metal atomic centers offers a practical and feasible strategy for regulating the electronic structures and thus improving the intrinsic ORR activity of carbon-based SMACs.

## Regulation of the extended local coordination structure

In addition to the local coordination structure (i.e., metal atomic centers and the first coordination shell), the extended local coordination structure (i.e., the second and higher coordination shells) also can be regulated to further optimize the electronic structure of carbon-based SMACs and improve the intrinsic ORR performance. Generally, the coordination atoms in the second and higher shells refer to the atoms that are not directly connected with the metal atomic centers. Theses coordination atoms could affect the electronic structure of carbon-based SMACs through long-range delocalization of electron and exhibit moderate influence on electrocatalytic ORR activity. To date, both heteroatom incorporating and vacancy engineering have been proposed to tailor the extended local coordination structure. In this section, we briefly summary the recent progresses in regulating the second and higher coordination shells of various carbon-based SMACs for high-performance electrocatalytic ORR.

### Heteroatom incorporating

**Fig. 8:**
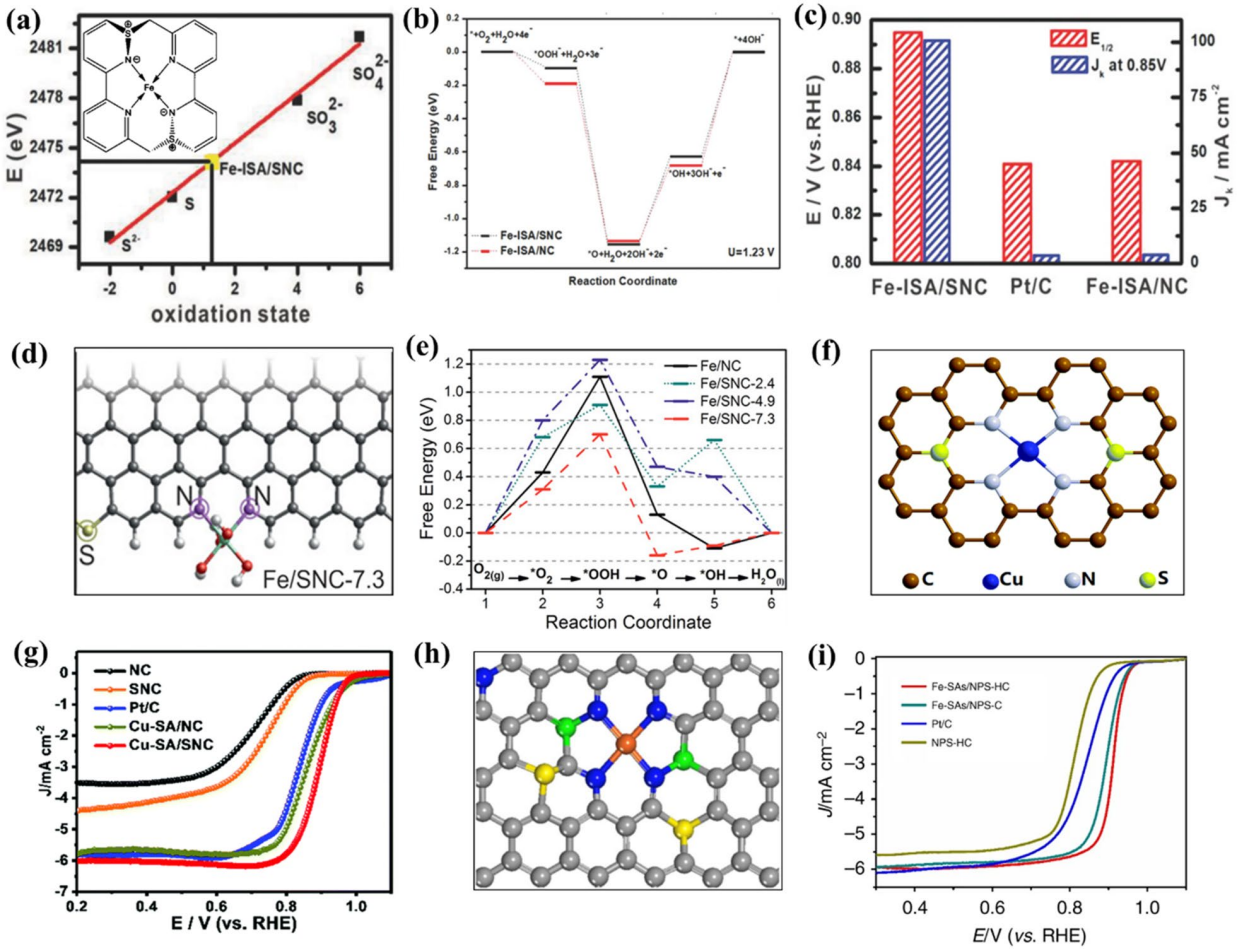
**a** Linear fitting curve of oxidation state for Fe-ISA/SNC and reference materials. The insert is the molecular structure of Fe-N_4_S_2_ active site in Fe-ISA/SNC. **b** Free energy diagrams of ORR on Fe-ISA/SNC and Fe-ISA/NC. **c** Comparison of J_k_ at 0.85 V and E_1/2_ of Fe-ISA/SNC, Fe-ISA/NC, and Pt/C. **d** Geometric structure of Fe/SNC-7.3. **e** Free energy diagram of ORR on Fe/NC and Fe/SNC. **f** Schematic interfacial model of Cu-SA/SNC. **g** LSV curves of Cu-SA/SNC, Cu-SA/NC, SNC, NC, and Pt/C catalysts in O_2_-saturated 0.1 M KOH solution. **h** Schematic model of Fe-SAs/NPS-HC, Fe (orange), N (blue), P (green), S (yellow) and C (gray). **i** LSV curves of Fe-SAs/NPS-HC, Fe-SAs/NPS-C, NPS-HC, and Pt/C catalysts in O_2_-saturated 0.1 M KOH solution. **a**-**c** Reproduced with permission from Ref. [119]. Copyright 2018, Wiley-VCH. **d**, **e** Reproduced with permission from Ref. [115]. Copyright 2017, Wiley-VCH. **f**, **g** Reproduced with permission from Ref. [118]. Copyright 2019, Royal Society of Chemistry. **h**, **i** Reproduced with permission from Ref. [32]. Copyright 2018, Nature Publishing Group

Incorporating of non-metallic heteroatoms (e.g., S, P, B, etc.) into carbon-based SMACs has been recently developed as a feasible and effective approach to tune the second and higher coordination shells. As above mentioned, the incorporated heteroatoms outside the first coordination shell could affect the distribution of electron density over the metal atomic centers and thus modulating the intrinsic ORR activity [115–118]. Among various heteroatoms, S has been most widely studied and considered to be a highly effective heteroatom in tailoring the extended local coordination structure of carbon-based SMACs. Generally, S has a relatively lower electronegativity (2.58) than that of N (3.04), which could leads to an apparent polarization of N-S bonds (i.e., electrons shift from S to N). Therefore, the bonding of S with the N atom of M-N_4_ in the first coordination shell could improves the electron density of the metal atomic center and thus affects the electrocatalytic ORR performance. For instance, Li et al. reported that the incorporation of S atom in the second coordination shell of carbon-based Fe-N_4_ catalyst resulted in significant electron redistribution from S to N atom in the first coordination shell, and thus a positive valence state of S (+ 1.2) (Fig. 8a) [119]. DFT calculations demonstrated that the reduction of *OH to OH^−^ is the potential limiting step for both S-free Fe-N_4_ (Fe-ISA/NC) and S-incorporated Fe-N_4_ (Fe-ISA/SNC) catalysts, while the S-incorporated Fe-N_4_ catalyst is more thermodynamically favourable for this key step compared with the S-free Fe-N_4_ catalyst (Fig. 8b). This can be attributed to the S atom-induced negative charge accumulation on N atoms in the first shell which facilitates the *OH desorption and the enhancement of electrocatalytic ORR activity. As a result, the S-incorporated Fe-N_4_ catalyst exhibited more positive *E*_*1/2*_ of 0.896 V and larger kinetic current density (*J*_*k*_) of 100.7 mA cm^− 2^ at 0.85 V compared to the S-free Fe-N_4_ catalyst (0.839 V and ~ 3.60 mA cm^− 2^, respectively) (Fig. 8c). Besides, the bonding of S with the C atoms in the vicinity of the M-N_4_ center also could promotes the electrocatalytic ORR performance. For example, Chen and co-workers observed improved electrocatalytic ORR activity of S-incorporated Fe-N_4_ catalyst and attributed the boosted activity to the positively charged carbon atoms induced by the uneven charge distribution after bonding with S atoms which favour the adsorption of oxygen species [116]. In addition, Shen et al. figured out that the position of incorporated S atoms plays a crucial role in boosting the electrocatalytic ORR activity of carbon-based SMACs [115]. Based on their in-depth theoretical calculations, the authors demonstrated that the promoting effect of incorporated S atom on the intrinsic ORR activity only can be accessed when the S atoms were situated at least 7.3 Å away from the Fe atomic center which anchored at the zigzag edge of carbon matrix (Fig. 8d). In sharp contrast, shorter distances of 2.4 Å and 4.9 Å between S atom and Fe center did not benefit the electrocatalytic ORR process and afforded similar or even larger overpotentials in comparison to the S-free catalyst (Fig. 8e). In addition to metal Fe atomic center, Jiang and co-authors recently reported that the incorporation of S atom in the extended local coordination structure of carbon-based Cu-N_4_ catalyst could improve the intrinsic ORR activity (Fig. 8f) [118]. The synthesized S-incorporated Cu-N_4_ catalyst displayed outstanding electrocatalytic ORR activity with a much higher *E*_*1/2*_ of 0.893 V than that of the S-free Cu-N_4_ catalyst (0.862 V) (Fig. 8g). Theoretical calculations revealed that the atomic interface effect induced by the incorporated S atom resulted in less positively charged Cu center which is beneficial to the enhancement of electrocatalytic ORR performance.

In addition to S, P and B can also be employed as effective heteroatoms to tailor the extended local coordination structure of metal atomic centers in carbon-based SMACs. Similar to S, the incorporated P atom in carbon-based Fe-N_4_ catalyst also has been verified to display electron-donating properties due to its low electronegativity (2.19). As reported by Chen and co-workers, the electron donation from P atom that bonded with the N atom of Fe-N_4_ center in the first coordination shell could results in less positively charged metal Fe center, thereby reducing the *OH binding energy during the electrocatalytic ORR [32]. However, in contrast to S and P heteroatoms with electron-donating properties, the bonding of electron-withdrawing B atom with the N atom of Fe-N_4_ center in the first coordination shell generally results in the shifting of electrons from N to B. For instance, Sun and co-workers reported that the charge density redistribution induced by the bonding of B atom with the N atom of Fe-N_4_ in the first coordination shell could modulate the d-band center of the metal atomic center in carbon-based SMACs to render favourable adsorption energy of oxygen and thus higher intrinsic ORR activity [120]. A similar work reported by Yuan and co-authors also observed the enhanced intrinsic ORR activity of carbon-based Fe-N_4_ catalyst by incorporating B atom with electron-withdrawing properties to regulate the extended local coordination structure [121]. Furthermore, the extended local coordination structure of metal atomic centers in carbon-based SMACs can also be synergistically regulated by simultaneously incorporating different kinds of heteroatoms. For instance, Chen et al. reported that the simultaneous incorporation of S and P heteroatoms into the carbon-based Fe-N_4_ catalyst renders a superior electrocatalytic ORR activity in comparison to the Fe-N_4_ catalysts with no heteroatom or a single heteroatom (Fig. 8h, i) [32]. The authors attributed the increase in intrinsic ORR activity to the synergistic electron donation from both S and P atoms to the metal Fe center, which consequently leads to the weakening of the *OH binding. Overall, based on the above discussion, the key point to optimize the extended local coordination structure in carbon-based SMACs lies in the appropriate regulation of electron density over the metal atomic centers, which could reduce the energy barrier for crucial steps during the electrocatalytic ORR process.

### Defect engineering

**Fig. 9:**
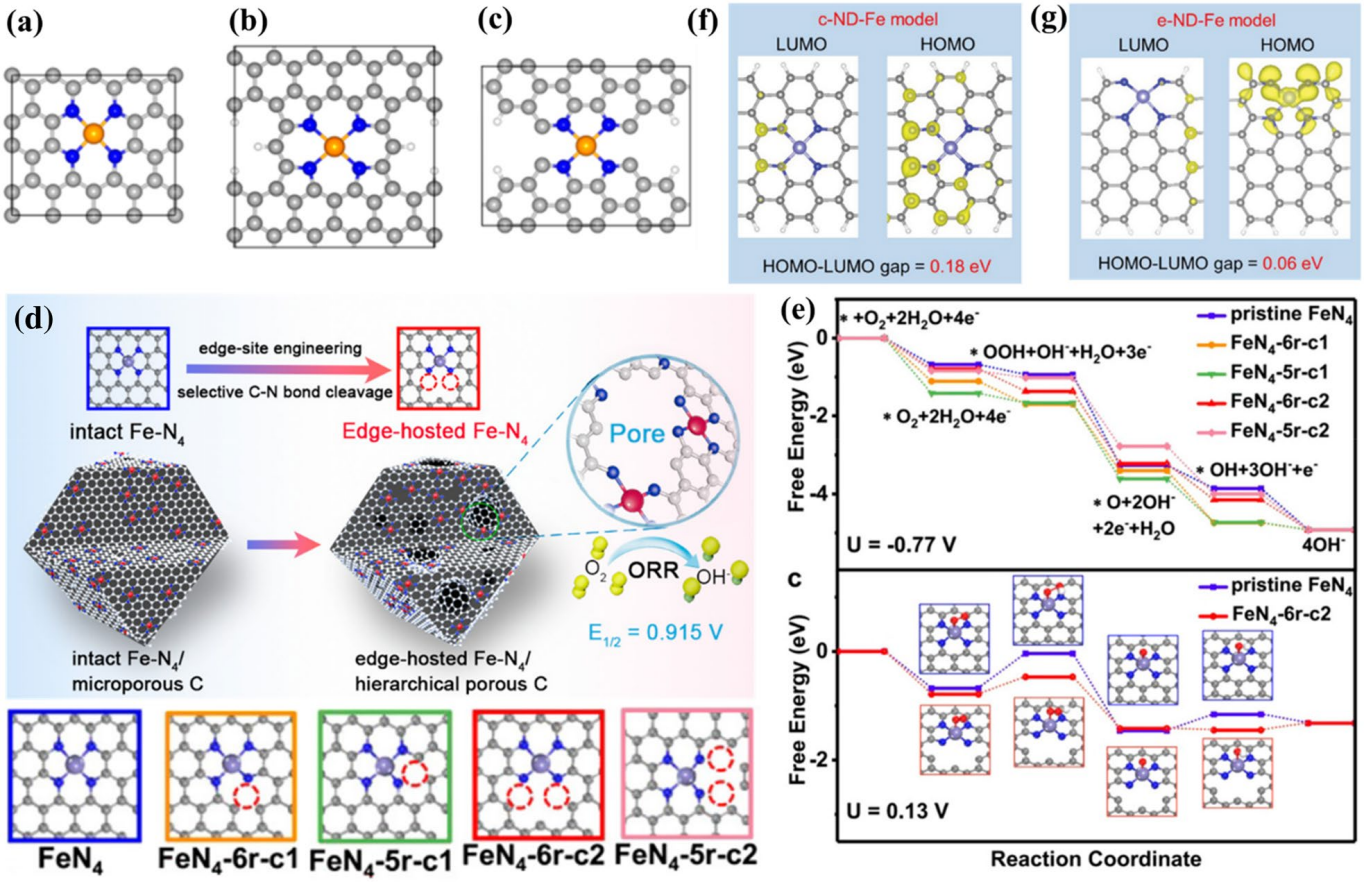
**a**–**c** Atomistic structures of Fe-N_4_ sites with different extended local coordination structures: **a** Fe-N_4_-C_10_, **b** Fe-N_4_-C_12_, and **c** Fe-N_4_-C_8_. **d** Schematic of carbon-based Fe-N_4_ catalyst with abundant edge-hosted Fe-N_4_ sites for high-efficiency electrocatalytic ORR (top). Five possible atomic configurations of Fe-N_4_ sites with and without carbon defects (bottom). **e** Free energy diagrams of ORR on Fe-N_4_ sites with different configurations at U = -0.77 V and U = 0.13 V. **f**, **g** Highest-occupied molecular orbital (HOMO) and lowest-unoccupied molecular orbital (LUMO) distributions of the c-ND-Fe (**f**) and e-ND-Fe (**g**) sites. **a**-**c** Reproduced with permission from Ref. [104]. Copyright 2017, American Chemical Society. **d**, **e** Reproduced with permission from Ref. [122]. Copyright 2018, American Chemical Society. **f**, **g** Reproduced with permission from Ref. [124]. Copyright 2020, Wiley-VCH

Apart from heteroatom incorporating, the electronic structure of carbon-based SMACs can also be regulated via constructing carbon defects in the extended local coordination structure of the metal atomic centers. The constructed carbon defects have been acknowledged to enhance the intrinsic ORR activity of carbon-based SMACs through the optimization of free energies for the reaction intermediates over the metal atomic centers. For instance, Liu et al. studied the effect of carbon defects near the Fe-N_4_ sites in carbon-based Fe-N_4_ catalysts on electrocatalytic ORR activity by employing DFT calculations [104]. The authors figured out that the Fe-N_4_ sites at the edge of the micropores (Fe-N_4_-C_8_) displayed the best electrocatalytic ORR activity among the three Fe-N_4_ catalysts with different extended local coordination structures (Fig. 9a–c). The superior intrinsic ORR activity of Fe-N_4_-C_8_ sites can be attributed to their lowest activation energy (~ 0.20 eV) for the breaking of O-O bond in *OOH intermediate and consequently promoted four-electron pathway of electrocatalytic ORR. This work suggests that the rational construction of carbon defects in the extended local coordination structure of the metal atomic centers could enhance the intrinsic ORR activity of carbon-based SMACs. Following this strategy, Jiang and co-authors reported the synthesis of carbon-based Fe-N_4_ catalyst with abundant edge-hosted Fe-N_4_ sites for high-efficiency electrocatalytic ORR (*E*_*1/2*_ of 0.915 V) under alkaline conditions (Fig. 9d) [122]. To gain deep insights into the intrinsic ORR activity of edge-hosted Fe-N_4_ sites, the authors investigated five possible atomic configurations of carbon defects. As exhibited in Fig. 9e, all the four-electron ORR processes were exothermic at an equilibrium potential of −0.77 V. However, the largest positive free energy change (ΔG) of the first electron transfer process (*O_2_ to*OOH) at 0.13 V indicated the rate-determining step. The FeN_4_-6r-c2 configuration displayed the most appropriate values of ΔG for each ORR step, thus possessing higher intrinsic ORR activity compared to the carbon defects-free Fe-N_4_ configuration. This work provides in-depth understanding of carbon defects effect on the intrinsic ORR properties of metal atomic centers. Furthermore, Fu and co-workers synthesized a carbon-based Fe-N_4_ catalyst with a highly porous structure via a simple NH_4_Cl-assisted pyrolysis approach [123]. A high fraction of Fe-N_4_ sites were verified to be preferentially hosted by the graphene-like porous structures. DFT calculations demonstrated that the neighbouring carbon defects could significantly improves the *O_2_ adsorption and facilitates the four-electron pathway of electrocatalytic ORR. In addition, Wang and co-workers reported the construction of edge nitrogen-modified divacancies trapped Fe-N_4_ (e-ND-Fe) sites as a highly efficient catalyst for electrocatalytic ORR [124]. DFT calculations suggested that the e-ND-Fe sites could induce a more pronounced local electronic redistribution than carbon defects-free Fe-N_4_ sites, which consequently results in higher electron density around the Fe-N_4_ sites and a narrower bandgap (Fig. 9f,g). As a result, a lower free-energy barrier toward direct four-electron pathway for electrocatalytic ORR can be realized. In addition to the Fe-N_4_ sites, the defect engineering is also applicable to the Co-N_4_ sites. For example, He and co-workers found that the Co-N_4_ sites surrounded by carbon defects are capable of catalyzing oxygen reduction in a four-electron pathway under acidic conditions and thus exhibiting comparable ORR activity to the Fe-N_4_ sites, in sharp contrast to the conventional Co-N_4_ sites with high two-electron pathway selectivity [125]. Overall, the rational defect engineering offers a new pathway to tailor the extended local coordination structure and improve the intrinsic ORR activity of carbon-based SMACs.

## Summary and perspective

Carbon-based SMACs have emerged as one of the promising candidates to be used as ORR catalysts in renewable energy conversion technologies owing to their impressed electrocatalytic ORR performance. Recent advances have manifested that the coordination structure in carbon-based SMACs governs the electronic structure and thus the intrinsic electrocatalytic ORR activity. Accordingly, massive research efforts have been devoted to developing advanced carbon-based SMACs for high-efficiency electrocatalytic ORR via regulating the coordination structure. Specifically, the strong electronic hybridization between the metal atomic centers and adjacent coordination atoms in the first shell causes significant electron density redistribution of the local coordination structure, which provides a promising approach to regulate the valence state and modulate the strength of chemical adsorption for ORR-relevant species. In addition, the heteroatoms and defects incorporated in the extended local coordination structure also moderately modulate the electronic structures of carbon-based SMACs, whereby the adsorption energies of ORR-relevant species over the metal atomic centers can be optimized. In this review, the crucial role of coordination structure including the local coordination structure (i.e., metal atomic centers and the first coordination shells) and extended local coordination structure (i.e., the second and higher coordination shells), on the intrinsic ORR activity of carbon-based SMACs have been critically examined. The intrinsic structure-activity relationships between recently reported coordination structures and electrocatalytic ORR activities were discussed and paid particular attention for better understanding the electrocatalytic ORR mechanisms of carbon-based SMACs. Recent progresses regarding coordination regulation for the rational design of advanced carbon-based SMACs for electrocatalytic ORR were emphatically exemplified and discussed.

Despite considerable progress has already been achieved in this field, however, some critical issues regarding the rational regulation of the coordination structure in carbon-based SMACs to enable their practical applications have not yet been well-solved. Here, we would like to highlight the following some urgent challenges and possible future directions: (1) More attention is required to be paid on the precise synthesis of carbon-based SMACs at the atomic level to obtain the desired well-defined coordination structure. Currently, most carbon-based SMACs are generally prepared through a high-temperature pyrolysis treatment of the pre-designed precursors, thus possessing unpredictable and poorly defined multiple coordination structures and hindering the insightful understanding of structure-activity relationships. In this regard, the development of pyrolysis-free synthesis routes is of great interest to enable well-preserved active sites and gaining deep insights into the electrocatalytic ORR mechanisms. (2) The precise identification of coordination structure still remains a great challenge, since the currently widely employed analysis techniques (e.g., synchrotron-based X-ray absorption fine structure (XAFS)), tend to yield only average coordination structure. Therefore, the integrated utilization of multiple characterization technologies (e.g., aberration corrected transmission electron microscopy, electron energy loss spectroscopy, X-ray photoelectron spectroscopy, XAFS, DFT calculations, etc.) is currently necessary to achieve a relatively accurate coordination structure. Great effort should be definitely devoted to developing more advanced characterization techniques to access the precise coordination structure of carbon-based SMACs. (3) The dynamic evolution of coordination structure in some cases during the electrocatalytic ORR process, which significantly affects the structure-activity relationships, has not attracted enough attention. Therefore, although some are still challenging in analytical accuracy and technical feasibility, various operando/in situ techniques are strongly recommended in future studies to monitor the dynamic structure and deepen the insightful understanding of electrocatalytic ORR mechanisms. (4) Despite the intrinsic ORR activity of carbon-based SMACs has been upgraded greatly through coordination regulation, their performance in practical applications has not yet been improved substantially. Other important aspects, such as metal-atom loadings, physicochemical properties of the carbon matrixes, mass transfer and durability, which also paly crucial roles in practical ORR applications should be considered concurrently in the rational design of carbon-based SMACs. (5) Given that the current general electrochemical measurements are for the entire electrode, the experimental evaluation of intrinsic ORR activity for carbon-based SMACs remains very challenging. Future efforts should be made to reveal more universal principles regarding the relationships between coordination structure and intrinsic ORR activity by taking advantage of advanced microelectrochemical measurements and microscopic imaging techniques with atomic resolution.

Overall, remarkable progress has been made in the development of high-performance carbon-based SMACs for electrocatalytic ORR via rational coordination regulation. New opportunities for the exploration of proper coordination structure of carbon-based SMACs toward electrocatalytic ORR are still abundant. We anticipate that this review will propel the development of advanced carbon-based SMACs for electrocatalytic ORR and provide valuable information to help overcome the grand challenges in developing more efficient renewable energy conversion technologies.

## Data Availability

Not applicable.
